# Mesenchymal stromal cell isolation from pond slider (*Trachemys scripta*) adipose tissue obtained during routine neutering: a model for turtle species

**DOI:** 10.3389/fvets.2025.1546091

**Published:** 2025-03-19

**Authors:** Valentina Andreoli, Alessandro Vetere, Virna Conti, Martina Gavezzoli, Priscilla Berni, Roberto Ramoni, Giuseppina Basini, Giordano Nardini, Igor Pelizzone, Stefano Grolli, Francesco Di Ianni

**Affiliations:** ^1^Department of Veterinary Medical Science, University of Parma, Parma, Italy; ^2^Clinica Veterinaria Modena Sud, Modena, Italy

**Keywords:** mesenchymal stromal cells, regenerative medicine, veterinary regenerative medicine, chelonia, reptile medicine, turtles, tortoise

## Abstract

**Introduction:**

Mesenchymal stromal cells (MSCs) hold great clinical potential in veterinary regenerative medicine. However, a notable gap exists in the literature regarding the isolation and characterization of these cells in reptiles. The objective of this study was to evaluate the feasibility of isolating adipose tissue-derived mesenchymal stem cells (MSCs) from pond slider (*Trachemys scripta*) tissue samples collected during routine neutering procedures.

**Methods:**

Adipose tissue samples were obtained from five animals and processed using an enzymatic procedure. The resulting cell suspension was subsequently cultured at 28°C in a controlled atmosphere with 5% CO_2_. The cell growth rates were evaluated through direct counting of cells up to passage 7. The colony-forming unit (CFU) capacity of MSCs was evaluated in low-density cell cultures, and the ability of the cells to differentiate into adipogenic, chondrogenic and osteogenic lineages was assessed. The cell phenotype was characterized at the molecular level using reverse transcription-polymerase chain reaction (RT–PCR) and amplicon sequencing, with a focus on markers commonly used for gene expression profiling of mammalian MSCs.

**Results:**

The cells demonstrated the capacity to differentiate into adipogenic, chondrogenic, and osteogenic lineages. RT–PCR revealed the expression of CD105, CD73, CD44, and CD90, whereas CD34 and HLA-DRA were not expressed. Sequence homology analysis demonstrated that the amplicons matched the sequences reported in the *Trachemys scripta* whole-genome shotgun sequence. This study represents the first investigation aimed at the isolation, *in vitro* expansion, and characterization of reptile adipose tissue-derived MSCs.

**Discussion:**

The results demonstrate the feasibility of isolating MSC-like cells from chelonian adipose tissue and underscore their potential for application in regenerative medicine for both companion reptiles and endangered wild species.

## 1 Introduction

Mesenchymal stromal cells (MSCs) are multipotent cells that can be isolated and expanded from a range of tissues, including bone marrow, adipose tissue, the umbilical cord, and the placenta (1). Initially investigated as pro-regenerative therapeutic agents based on their capacity to differentiate into multiple cell types, including osteoblasts, chondrocytes, and adipocytes, they have since been evaluated primarily for their immunomodulatory potential (2, 3). MSCs have the capacity to modulate immune responses, reduce inflammation, and promote tissue healing through paracrine signaling, rendering them an appealing option for the treatment of a multitude of diseases (4). In veterinary medicine, MSCs have been applied in the treatment of musculoskeletal diseases, as well as in organ and systemic pathologies where acute and chronic inflammation processes play pathogenic roles. A non-exhaustive list of veterinary pathologies investigated with MSCs includes orthopedic and musculoskeletal diseases (osteoarthritis, tendon and ligament injuries, desmopathies), neurological diseases (spinal cord injuries, degenerative myelopathy) and immune-mediated and inflammatory diseases (canine and feline inflammatory bowel disease, feline chronic gingivostomatitis, atopic dermatitis, chronic kidney disease) (5). Additionally, MSCs have been utilized in the management of ophthalmic and cardiac diseases, as well as chronic non-healing wounds (5). One of the most noteworthy characteristics of MSCs is their capacity to serve dual roles in both regenerative and immunomodulatory therapies. They are capable of secreting a diverse array of bioactive soluble molecules, including immunomodulatory cytokines and growth factors, and producing extracellular vesicles (EVs), which facilitate the modulation of the local immune environment, promote healing, and prevent excessive scarring (3). MSCs are isolated and expanded from a variety of tissues, including bone marrow, adipose tissue, peripheral venous blood, endometrium, skin, dental pulp, and perinatal tissues such as the umbilical cord, amniotic membranes, and fluid. Adipose tissue and bone marrow represent the most extensively investigated sources of cells for clinical application, offering an accessible route for the isolation of autologous MSCs from adult patients (1, 6).

Although the literature has documented the use of blood-derived products such as thrombocyte–leukocyte-rich plasma (TLRP) as adjunctive regenerative therapies for various lesions in reptiles (7–9), there is a significant gap in research concerning the isolation and characterization of MSCs within this taxonomic class. This study represents the first attempt to isolate, expand, and characterize MSCs derived from reptilian adipose tissue. The feasibility of isolating turtle MSCs with characteristics similar to those described for mammalian species was explored starting from adipose tissue samples collected during routine *Trachemys scripta* neutering. When possible, the cells were characterized using the methods described for isolation from species of veterinary interest. The objective of this study was to evaluate the possibility of applying MSC-based regenerative medicine protocols to companion reptiles and endangered wild species.

## 2 Materials and methods

### 2.1 Ethics statement, species, and animal selection criteria

Ethical approval for the study was given by the University of Parma ethical committee (PROT. No. 15/CESA/2022). Five clinically healthy adult female pond sliders (*Trachemys scripta*) aged 20–30 years were presented to the Department of Veterinary Science, University of Parma, Italy, for routine spaying. *T. scripta* is not a reptile species of relevant clinical interest in the veterinary clinic but was used in the present study as a model reptile. In Italy, *T. scripta* is considered an alien invasive species because it competes with native species for food resources and habitats. To manage the *Trachemys scripta* population, a national control plan (National Plan for the Management of the American Marsh Tortoise (*Trachemys scripta*) 2020 [Decree of the Minister for Ecological Transition No. 370 of September 28, 2022 (10)] has been approved that requires the sterilization or culling of individuals in the territory (11, 12). The study involved the enrolment of subjects at the University of Parma Teaching Veterinary Hospital (OVUD) by the local ENPA, an Italian national association for the protection of animals that have been rescued from various situations (abandonment, found in parks, and public places). During the sterilization process, peri-ovarian adipose tissue samples were recovered. The tissue would have otherwise been discarded as a consequence of the sterilization procedure. No patients underwent surgery with the specific purpose of harvesting adipose tissue. The animals were weighed with an analytical balance (VEVOR^®^ Digital Precision Scale 5,000 g × 0.01 g, Taicang Vevor Industry Co., Ltd. 9448 Richmond Pl, Rancho Cucamonga, CA, 91730, USA) from 1 to −23 kg [2.20 to −50.70 lb; median, 1.68 g (3.70 lb)] and measured from 22 to −28 cm (8.66 to −11.02 inches) [median 25.9 cm (10.19 inches)] along the curved carapace length (CCL). All the subjects underwent blood sampling from the jugular vein (13) of ~0.5 mL (1% live weight) (14). The sample was collected in tubes containing lithium heparin as an anticoagulant and used to perform a microhaematocrit for the evaluation of packed cell volume (PCV%), white blood cell count (WBC) using the Natt and Herrick stain method (15) and biochemical examination of the plasma prior to surgery to quantify analytes such as blood urea nitrogen (BUN), K+, total protein (TP), bile acids (BAs) and uric acid (UA). Exclusion criteria: For each animal, a dorsoventral X-ray projection was performed. Animals with eggs, a PCV < 20% or >35%, a WBC >19 × 10^3^/μL, and a TP < 1.5 or >8 g/dL were excluded from the study (15). Ultimately, no animals were excluded.

### 2.2 Surgical procedure

**Figure 1:**
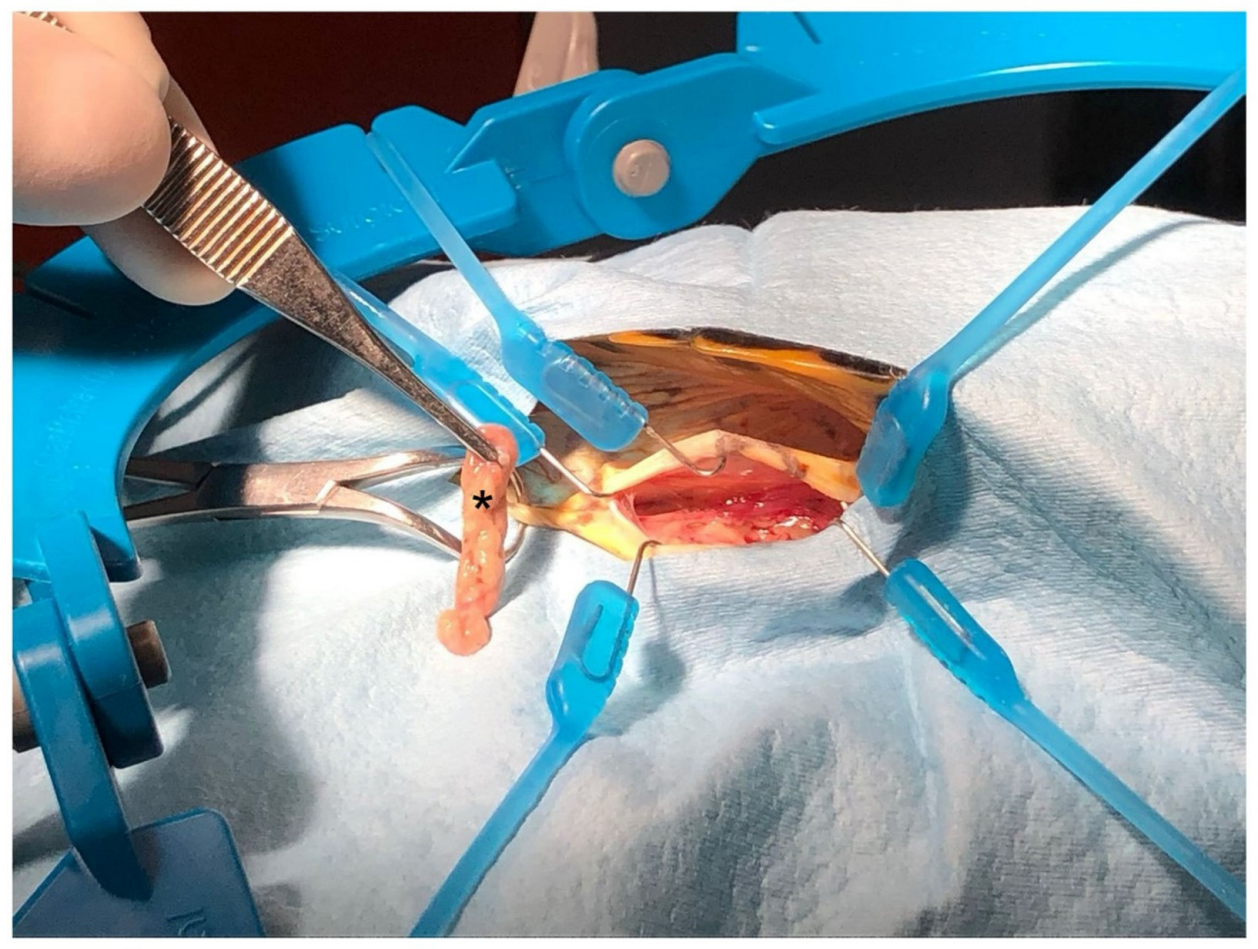
Collection of adipose tissue samples (fat pads of the prefemoral fossa) from *Trachemys scripta* during neutering surgery. Note the brownish colouration of the tissue (asterisk).

The animals were hospitalized 1 week before surgery, housed individually in plastic containers, and acclimatized with light and heat from 50-W metal halide lamps (Reptifood S.r.l., Roma, Italia). Food was withheld 48 h before surgery. Room temperature was maintained between 25 and 28°C, and body temperature was monitored using a cloacal digital thermometer and maintained at 28°C. Each animal was identified with a unique number, and candidates were selected for surgery through simple randomization using randomizer software (16). A combination of ketamine (10 mg/kg) (Acme S.r.l., Milano, Italy), dexmedetomidine (50 μg/kg) (Vétoquinol Italia S.r.l., Bertinoro, FC, Italy), and midazolam (1 mg/kg) (Hameln Pharmaceuticals, Gloucester, UK) was administered intramuscularly in the left forearm. For multimodal analgesia, meloxicam (0.2 mg/kg) was given 1 h before anesthesia (17, 18). A venous catheter was placed in the jugular vein for access, and the subjects were monitored with Doppler, ECG, and capnography to assess anesthesia depth. The eyelid reflex, mandibular tone, neck extension, and limb retraction were checked every 5 min to evaluate analgesia and recovery. Loss of limb retraction indicated adequate anesthesia, whereas mandibular tone loss allowed intubation. In the event of failure to lose mandibular tone, propofol was used as an induction agent at 5 mg/kg (18, 19). During surgery, the subjects were maintained on halogenated gases (1.5% isoflurane, Zoetis Italia S.r.l., Rome, Italy) and manually ventilated at 4 breaths per min. At the end of the procedure, dexmedetomidine was antagonized with atipamezole (0.5 mg/kg) intramuscularly (20). The subjects were positioned in left lateral recumbency and secured with foam support and tape, with their hind limbs extended and taped together to expose both flank fossae. A surgical scrub was performed on the prefemoral fossa and adjacent areas. A 2 cm cranio-caudal incision was made in the center of the prefemoral fossa using a no. 11 blade (Mealli S.r.l., Firenze, Italy), followed by subcutaneous dissection. Approximately 0.5–1 g of adipose tissue was aseptically collected from each animal before it reached the aponeurosis of the abdominal muscles for coelomic access (Figure 1). The right ovary and oviduct were identified using a 30° oblique telescope with an operative sheath (Endoscopia Italia S.r.l., Verona, Italy) and then exteriorized with sterile swabs. Vessels of the ovarian pedicle, mesovarium, and mesosalpinx were cauterized and excised with an electrocoagulation device (EnSeal^®^, Alcyon Italia, Rome, Italy). The oviduct was ligated ~3 cm from the cloacal opening and removed. The muscle layers were closed with simple continuous sutures, and the skin layers were closed with interrupted sutures, both of which used a 2/0 absorbable monofilament (Braun Avitum Italy S.p.A. Mirandola, Italy). The procedure was repeated on the opposite side. Finally, 0.5 mg/kg atipamezole (Fatro, Ozzano dell'Emilia, Italy) was administered as a reversal agent (21), along with a single 10 mg/kg dose of tramadol IM and 0.2 mg/kg meloxicam SC once daily for 5 days. No complications were observed during the surgical procedure or during recovery in any of the animals.

### 2.3 Histological evaluation of the adipose tissue sample

To confirm the actual nature of the obtained adipose tissue, a small fragment of the sample measuring ~0.5 cm^3^ was fixed with 10% neutral buffered formalin solution, dehydrated in a graded series of ethanol to absolute alcohol, cleared with xylene and embedded in paraffin. Five-micrometer-thick sections were stained with haematoxylin and eosin (22).

### 2.4 Isolation and culture of *Trachemys scripta* AD-MSCs (Ts-AD-MSCs)

The media, supplements, and other cell culture reagents used for cell culture were obtained from Gibco (Thermo Fisher Scientific Inc., Waltham, MA, USA) unless otherwise specified. The plastic labware used was from VWR (Avantor, Radnor, OR, USA).

### 2.5 Enzymatic digestion and setup of bidimensional and three-dimensional cultures

To isolate MSCs from the adipose tissue of *Trachemys scripta*, an enzymatic digestion protocol adapted from established mammalian methods was employed (23). The adipose tissue samples (0.56 ± 0.22 g) were briefly washed in 70% ethanol to remove bacterial contaminants and then transferred into phosphate-buffered saline (PBS), pH 7.4, to completely remove any residual ethanol. Before digestion, the tissue samples were mechanically minced into ~1 mm^3^ fragments using a no. 19 sterile surgical blade. The fragments were then placed in 15 mL Falcon tubes containing 5 mL of prewarmed DMEM enriched with 0.1% type I collagenase (w/v), and the tubes were placed at 37°C in an orbital shaking incubator set at 80 RPM for 1 h. After digestion, 5 mL of DMEM supplemented with 10% fetal bovine serum (FBS), penicillin (100 μg/mL), streptomycin (100 μg/mL), and amphotericin B (2.5 μg/mL) (maintenance DMEM, mDMEM) was added to the tubes to stop collagenase digestion, after which the tubes were centrifuged at 400 × g for 10 min. The supernatant was removed, and the cell pellet was resuspended in 1 ml of mDMEM. The cell suspension was then divided into two 0.5 mL aliquots. Each aliquot was seeded in a 3.5 cm diameter Petri dish containing 2 mL of mDMEM. The cell cultures were then placed in a dedicated incubator with a controlled atmosphere of 5% CO_2_ at 28°C. At the time of adipose tissue processing, small fragments were seeded into a three-dimensional gel to test the ability of the cells to migrate outwards from the tissue. Prior to collagenase digestion, the fragments were collected and seeded in a 3D gel composed of 10% calcium gluconate, 40% serum-free DMEM, and 50% plasma. Tissue fragments were cultured in a 3D environment exclusively to assess their viability. All characterization analyses of the obtained MSC-like populations were performed on cells obtained by digestion using the collagenase-based protocol.

### 2.6 Cell culture and amplification

The first medium change occurred 48 h after the seeding of the cell suspension. The maintenance DMEM was then replaced every 48 h to facilitate cell expansion. Once the cells reached ~80–90% confluence, they were detached with 0.3 mL of 0.05% trypsin-EDTA. Cell counting was performed manually at each passage using a Burker chamber. After counting, 8,000 cells/cm^2^ were seeded into two 25 cm^2^ cell culture flasks, which were maintained at 28°C in an atmosphere with 5% CO_2_. This process was repeated whenever the cells reached 90% confluence until passage 8.

### 2.7 Cryopreservation

After trypsinization, the cells were collected by centrifuging the cell suspension at 190 × g for 10 min. The supernatant was discarded, and the resulting pellet was resuspended in 1 mL of a freezing mixture composed of high-glucose DMEM supplemented with 50% FBS, penicillin/streptomycin (100 U/mL, 100 μg/mL), amphotericin B (2.5 μg/mL), and 10% dimethyl sulfoxide (DMSO). The cell suspension was then transferred into cryovials and placed in a freezing container (Mr Frosty, Fisher Scientific Italia, Segrate, Italy) at −80°C. The cells were then transferred to liquid nitrogen for long-term storage.

### 2.8 White blood cell isolation

White blood cells were used as a positive control to assess the expression of negative MSC markers (24). Two milliliters of venous blood was collected in sodium citrate from the caudal or cervical vein and processed within 1 h, following a protocol proposed by Di Ianni et al. in 2015 (9). Briefly, whole blood was carefully layered over a prewarmed Histopaque 1.077 solution at 28°C, avoiding mixing of the two fluids. The blood-to-histopaque volume ratio was maintained at 1:1. Each sample was then centrifuged at 150 × g for 20 min without using brakes. After centrifugation, the WBC cellular fraction was visually identified as a white ring at the interface with the Histopaque solution and was carefully collected to avoid contamination with the underlying layers. The cells were washed twice with 5 mL of sterile PBS and then centrifuged at 250 × g for 10 min. The resulting pellet was frozen at −80°C until it was used for gene expression analysis.

### 2.9 *In vitro* expansion potential

The growth curves of adipose tissue samples from five different individuals were analyzed to assess the *in vitro* expansion potential of the isolated cells. The evaluation of cellular growth parameters was conducted from passage 1 (P1) to passage 7 (P7). In our experimental setup, the seeding of the cell population freshly isolated from the tissue was considered P0. P1 indicates the first passage of the cells, meaning the first time they were detached, split, and reseeded into new flasks.

For each passage, 8,000 cells per square centimeter (8,000 cells/cm^2^) were seeded into two 25 cm^2^ flasks. Once the cells reached a semiconfluent state (80–90%), they were trypsinized and counted. The cells were then reseeded at a density of 8,000 cells/cm^2^, and this process continued through passage 7 (P7). The cell-doubling number (CDn) and cell-doubling time (DT) were calculated as suggested by Vidal et al. (25) and Roth (26). The two parameters were evaluated as follows: cell doubling number (CDn): ln (N_f_/N_i_)/ln2, and doubling time (DT): CT/CDn.

### 2.10 Colony-forming unit (CFU) assay

The CFU assay was performed on five different cell preparations at P2 and P5. For the CFU assay, 6-well plates were used; 100 and 200 cells/well were seeded in triplicate in mDMEM. The plates were incubated at 28°C with 5% CO_2_ for 14 days, and the DMEM was replaced every 72 h. After 14 days, the DMEM was removed, and the cells were fixed in 4% v/v formaldehyde for 1 h. The wells were subsequently stained using May–Grunwald Giemsa staining (22) to enable CFU counting. A CFU was defined as a cluster of at least 50 cells, and the counts were performed following the protocol described by Secunda et al. (27). Briefly, the wells were washed with distilled water, and 2 mL of May–Grunwald Giemsa stain (0.2 mL of Giemsa azure–eosin–methylene blue solution per mL of distilled water) was added for 12 min. After that, the wells were washed with distilled water, and colony counting was performed.

### 2.11 *Trachemys scripta* AD-MSC differentiation

Due to the lack of studies evaluating the differentiation capacity of adipose-derived cells in reptiles, protocols routinely used in mammals have been employed (27, 28). Differentiation assays were conducted to evaluate the potential for adipogenic, osteogenic, and chondrogenic differentiation. Each assay was performed in 6-well plates, with an initial seeding density of 6,000 cells/cm^2^. For each differentiation assay (*n* = 3; cell passage P3), a corresponding control was prepared, with a glass coverslip placed at the bottom of each well to facilitate cell staining and subsequent microscopic observation. To this aim glass coverslips were rinsed with distilled water and then immersed in a 0.1 N HCl solution for 2 h. Afterward, the coverslips were washed several times with distilled water and subsequently immersed in a 70% ethanol solution for 10 min. Finally, the coverslips were rinsed with sterile water and sterilized in an oven at 180°C for 2 h. Adipogenic differentiation treatment started once the cells reached ~70–80% confluence. The differentiation medium consisted of high-glucose DMEM supplemented with 10% FBS, antibiotics, and antifungal agents as described above and a mixture of 1 μM dexamethasone, 0.2 mM indomethacin, 0.5 μM IBMX (3-isobutyl-1-methylxanthine), and 1.7 μM insulin (10 μg/mL). This differentiation medium was maintained for 3 days, after which it was replaced with maintenance medium containing high-glucose DMEM supplemented with 10% FBS, antibiotics, antifungal agents, and 1.7 μM insulin (10 μg/mL) on the 4th day. On the 5th day, the medium was replaced with differentiation medium. This cycle continued until Day 24, when the cells were fixed with formalin and stained with oil red O (29). For chondrogenic differentiation, the StemPro^®^ Chondrogenesis Differentiation Kit (GIBCO) was used, with medium changes occurring every 3 days according to the manufacturer's guidelines. After the differentiation period (21 days), the cells were fixed in formalin and stained with Alcian blue (29). Osteogenic differentiation was induced when the cells reached 80–90% confluence using a medium consisting of high-glucose DMEM supplemented with 10% FBS, antibiotics, antifungal agents, 100 nM dexamethasone, 10 mM glycerophosphate, and 0.250 mM ascorbic acid. The medium was replaced every 48 h until Day 31. Following treatment, the cells were fixed with 95% methanol and stained with Alizarin red (29).

### 2.12 Gene expression analysis

To evaluate the phenotype of the cell populations, the expression levels of a panel of genes were evaluated by reverse transcription–PCR (RT–PCR), following the pattern of characterization suggested for mammalian cells (30). For total RNA extraction, the Macherey–Nagel NucleoSpin RNA Kit was used (31). RNA was extracted from P3 cell cultures (0.8–1 × 10^6^ cells) as well as from leukocytes isolated using a previously described procedure following the manufacturer's protocol. The total RNA obtained was quantified using the GeneQuant-pro RNA/DNA Calculator (Cambridge Scientific, Watertown, MA, USA), and 2 μg of RNA was reverse transcribed using a High-Capacity cDNA Reverse Transcription Kit (Applied Biosystems™, Cheshire, UK) following the manufacturer's guidelines. The primers used for RT–PCR were designed using the NCBI database with a dedicated software tool (Primer-BLAST, https://www.ncbi.nlm.nih.gov/tools/primer-blast/, accessed on March 18, 2024). The sequences were derived from the NCBI Reference Sequence database, based on the genome of *Trachemys scripta*, which was fully sequenced and published in 2020 (32). The primers, shown in Table 1, were synthesized by Macrogen Europe, Milan, Italy.

**Table 1:** Primer sequences, gene accession numbers, amplicon sizes, and Tm values of the primers.

**Gene**	**Primer sequence**	**GenBank accession number**	**Amplicon size (bp)**	**Tm**
CD44	Fw: GCTTCTACAGCCACCAGTTCTA Rv: CCACCCATCTTCTTGTCGGA	XM_034769691.1	263	59.77/59.39
CD73	Fw: CTTGGGAAATTTGATTTGTGATGCC Rv: GTAGACCACATGAATGCCTGC	XM_034766097.1	297	59.88/59.33
CD90	Fw: GAACAGCCTCAAGACCCGAT Rv: CCTTCCCTTCACAGGGACAC	XM_034754732.1	282	59.75/59.96
CD105	Fw: GTGCACAGACGACTCCATTG Rv: CACTCGAACGGCACCTCAA	XM_034793487.1	243	59.21/60.30
CD31	Fw: TGAGAAACTGCCATCTGGACC Rv: CTATTGGCAATGAGGAGGGGG	XM_034789735.1	278	60.00/60.20
CD34	Fw: GCAGAGTATTGAACGCCACC Rv: TCTGAGCCCCTCTGTTGAGA	XM_034770555.1	257	59.27/59.88
CD45	Fw: TTACGTGTCCGCTTTACGGG Rv: AGGAGACACCCTCACCTGTT	XM_034780075.1	269	60.39/60.10
HLA-DRA	Fw: CAGGGCGACTTCTACGACTG Rv: CCCCGCGTTTATAAGGGGC	XM_034789225.1	230	60.18/60.90
GAPDH	Fw: GAATGGGAAGCTCACTGGAA Rv: CCAAACTCATTGTCATACCAGG	XM_034787619.1	285	57.50/57.03

For gene expression analysis, nine genes were evaluated. Glyceraldehyde-3-phosphate dehydrogenase (GAPDH) was used as a housekeeping gene to validate the experimental results. CD44, CD73, CD90, and CD105 were used as positive markers for MSCs, whereas CD31, CD34, CD45, and HLA-DRA were used as negative markers. As a positive control for the expression of negative markers, cDNA from leukocytes was used (33). For RT–PCR master mix preparation, 1 unit of DreamTaq™ DNA Polymerase (Thermo Fisher Scientific, Baltics UABA V.A. Vilnius, Lithuania) was used, and 2 μg of cDNA, 1.125 μL per primer (from a 25 μM stock) and 1 μL of dNTPs (from a 10 mM stock) were added, resulting in a final reaction volume of 50 μL. Before sequencing, the presence of the expected amplicons was verified by 1.5% agarose gel electrophoresis. Amplicons were visualized using a UV transilluminator.

### 2.13 Sequencing

After confirming the expression of the individual genes by gel electrophoresis, an aliquot of each amplicon was sequenced using the Sanger sequencing service at Eurofins Genomics, Milan, Italy. The sequencing results were validated by comparing the obtained sequences with the specific sequences published on the NCBI website. The specific reference number for each gene is reported in Table 1. Sequence comparison was performed using the NCBI BLAST tool (https://blast.ncbi.nlm.nih.gov/Blast.cgi, accessed on September 16, 2024). A manual evaluation of the chromatographic traces from the sequencing was also conducted to confirm the results.

### 2.14 Statistical analysis

Statistical analysis was performed using the software Jamovi (version 2.5.6). Data from the cell proliferation assays were assessed for normality (Shapiro–Wilk test) and homogeneity of variance (Levene's test). Because the data were normally distributed, statistical analysis was conducted using one-way ANOVA, followed by Tukey's *post-hoc* test when resulted statistically significant (*p* ≤ 0.05). Data from the CFU counts were analyzed using a *t*-test. Given that these datasets were not normally distributed, a non-parametric Mann–Whitney *U*-test was applied for analysis.

## 3 Results

### 3.1 Histological evaluation of the samples and cell adhesion, migration, and CFU assays

**Figure 2:**
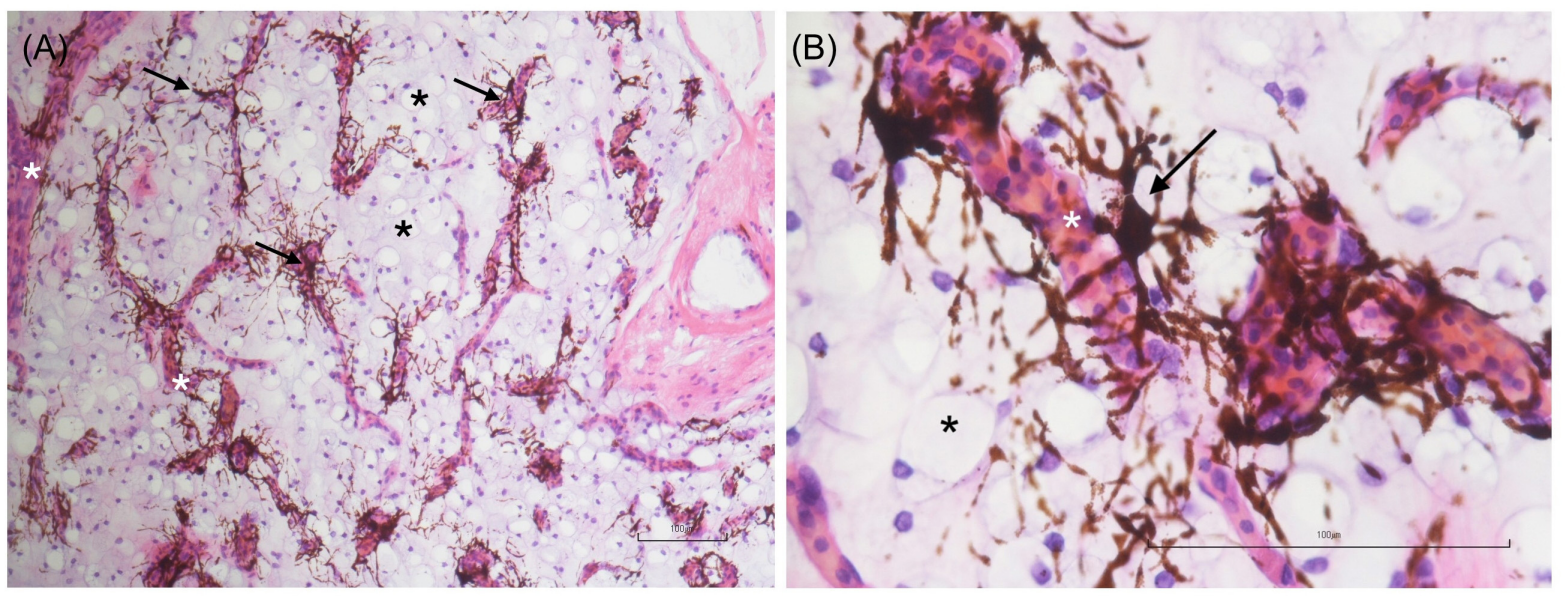
Low- **(A)** and high-magnification **(B)** images of adipose tissue sections stained with hematoxylin and eosin (H&E). Numerous mature adipocytes (black asterisks) and chromatophores (black arrows) arranged in a perivascular pattern are evident. The white asterisk indicates a blood vessel. Scale bar: 100 μm.

To confirm the actual nature of the adipose tissue samples, five-micrometer-thick sections were subjected to haematoxylin and eosin (H&E) staining. The analysis revealed numerous clusters of mature adipocytes associated with fibrous tissue, blood vessels, lymphatics, and perivascular chromatophores (Figure 2). After confirming the adipose tissue nature of the collected samples, the behavior of the cell population obtained following enzymatic digestion was evaluated.

**Figure 3:**
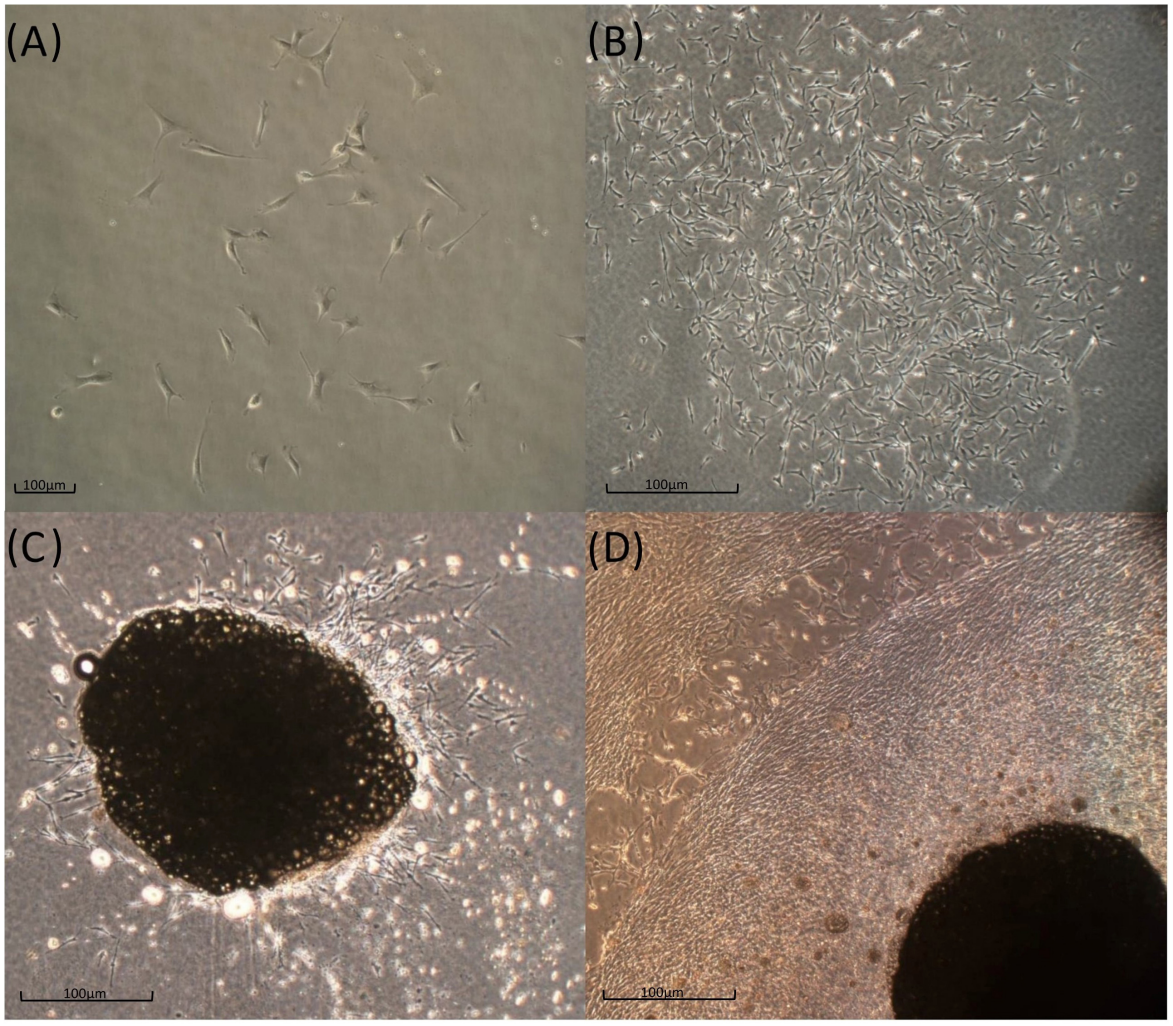
*In vitro T. scripta* MSC phenotype. Cells that were expanded in the 2D environment had a fibroblast-like appearance and tended to form small CFUs 5–7 days postseeding **(A)**, which gradually expanded in the following week **(B)**. Tissue fragments cultured in a 3D fibrin-derived environment show an outflow of fibroblast-like cells (**C**, Day 7) that gradually tend to occupy all the available space (**D**, 3 weeks).

**Figure 4:**
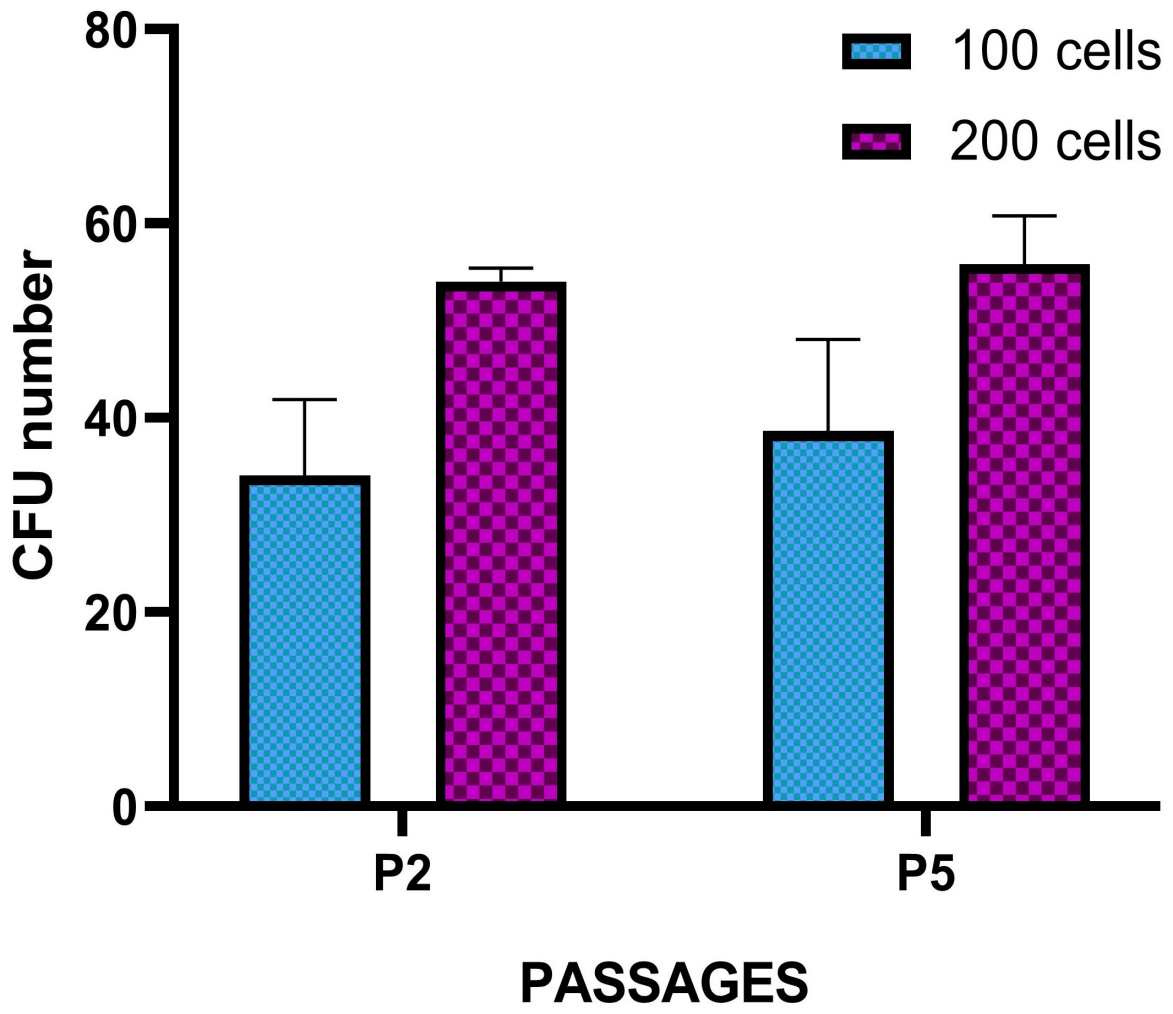
Colony-forming unit (CFU) assay of *T. scripta* MSCs. Colony formation was observed in MSCs seeded at 100 and 200 cells/well at P2 and P5. The results represent the mean ± standard deviation of three independent experiments. No significant difference was observed between P2 and P5 or between the different cell densities evaluated.

Spindle-shaped cells adhering to the culture plate surface were observed in all examined samples (Figure 3A) ~5–7 days after seeding the cellular pellets derived from the enzymatic digestion of adipose tissue. This morphology remained consistent throughout all the cell passages (P1 to P7). Over time, the adherent cells tended to form CFUs, eventually leading to full plate colonization (Figure 3B). In 3D cultures derived from the direct fragmentation and seeding of undigested adipose tissue, the first cellular entities began migrating from the fragments ~5–6 days postseeding (Figure 3C), fully colonizing the surrounding area in the following days. As shown in the image, the cells continued expanding until they encountered cellular populations from other fragments (Figure 3D). For the CFU formation assay, colony assessments were conducted at two different passages (P2 and P5). The average values obtained from seeding 100 and 200 cells across three different passages are shown in Figure 4. No statistically significant differences were observed in CFU counts between the two passages.

### 3.2 Growth curves

**Figure 5:**
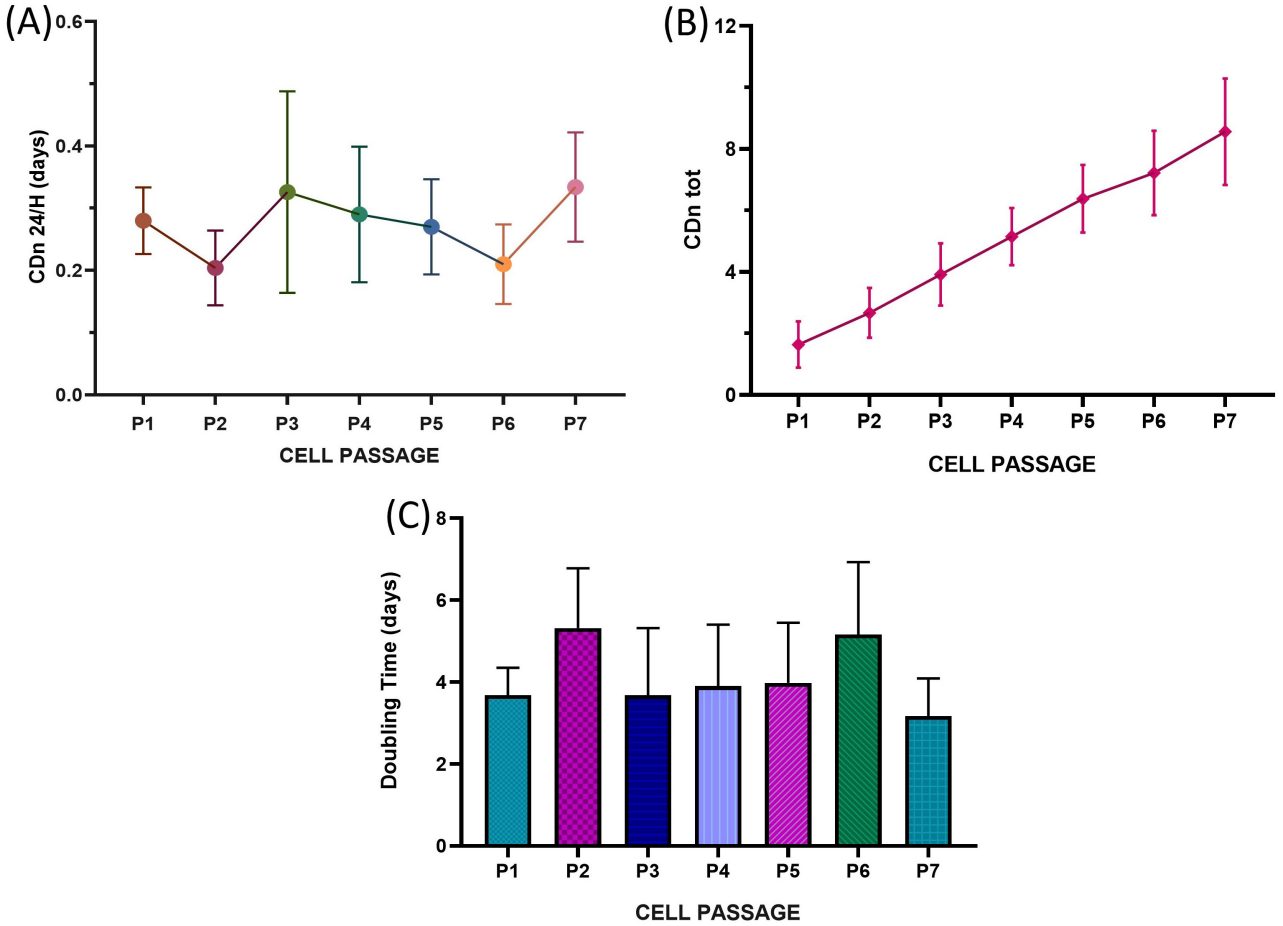
Long-term growth curves of *T. scripta* MSC-like cells. The fraction of cells that underwent cell replication at 24 h (CDn 24 h) **(A)**, total cell doubling number (CDn tot) **(B)** and mean doubling time (days, **C**) are reported from P1 to P7. No statistically significant differences were observed between the different cell passages.

Following the protocol described in Section 2.9, cell growth curves were evaluated by analyzing the growth curves of the five samples across successive passages, up to P7. For each passage, key parameters were evaluated, including the cell doubling number (CDn: ln (Nf/Ni)/ln2), the number of cell doublings per 24-h period (CDn/24 h), the cumulative number of cell doublings across all passages (total CDn: CDnP1 + CDnP2… + CDnPx), and the population doubling time (DT: CT/CDn). The average CDn/24 h, total CDn and DT values from passages P1–P7 were compared, and the results are reported in Figures 5A–C. Statistical analysis revealed no significant differences in cell growth rates between passages. Additionally, the consistent cell growth observed for total CDn (Figure 5B) suggested a continuous proliferation rate from P1–P7, with no plateau phase reached. Similarly, no statistically significant differences were observed in the doubling times (DTs, Figure 5C), further confirming the ability of these cell populations to sustain steady replication through the final passage tested. Furthermore, no statistically significant differences were identified in the parameters evaluated across the different animals considered.

### 3.3 Differentiation capacity of TS-ADMSCs

**Figure 6:**
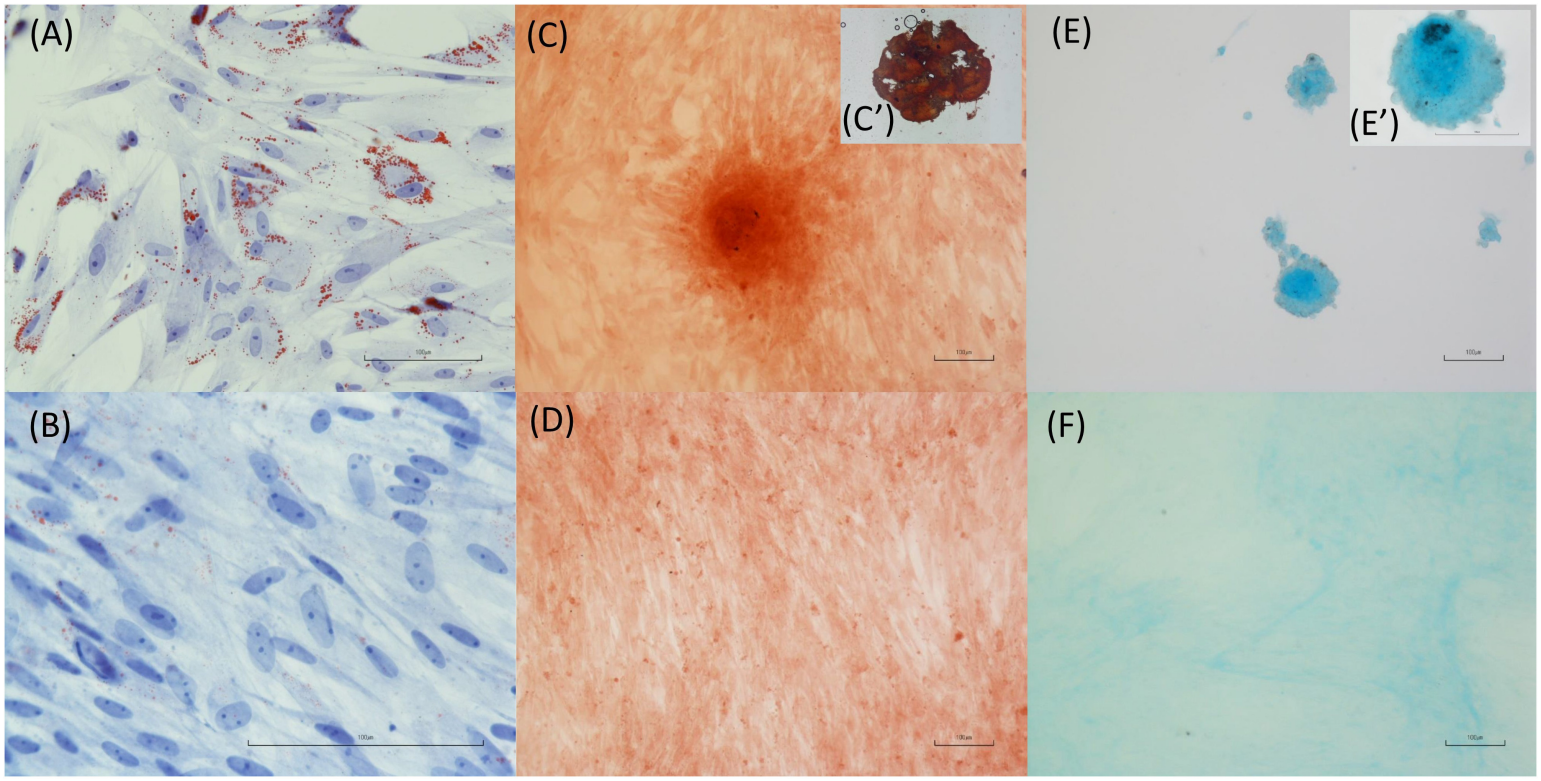
Differentiation capacity of *T. scripta* MSCs. Intracellular lipid accumulation (**A**, oil red O staining) is indicative of successful adipogenic differentiation. Osteogenic differentiation is supported by the staining of bone matrix-like deposits (**C**, **C′**, Alizarin red staining) in cells exposed to osteogenic medium. Finally, cartilage matrix formation **(E**, **E′)** is evidenced by Alcian blue staining of cell cultures treated with chondrogenic differentiation medium. Unstimulated cell cultures were used as a control **(B, D, F)**.

According to the differentiation protocols and staining procedures utilized, Ts-AD-MSCs are able to differentiate toward the adipogenic, osteogenic, and chondrogenic lineages. Figure 6A shows the accumulation of intracellular lipids, which is indicative of successful adipogenic differentiation. Figures 6E, E′ display the presence of cartilaginous matrix deposits positive for Alcian blue staining, indicating the chondrogenic differentiation of the MSCs. Figures 6C, C′ reveal bone matrix-like deposits, indicating the osteogenic differentiation of MSCs. A negative, unstimulated control was evaluated for each differentiation protocol (Figures 6B, D, F).

### 3.4 Gene expression analysis and sequencing

**Figure 7:**
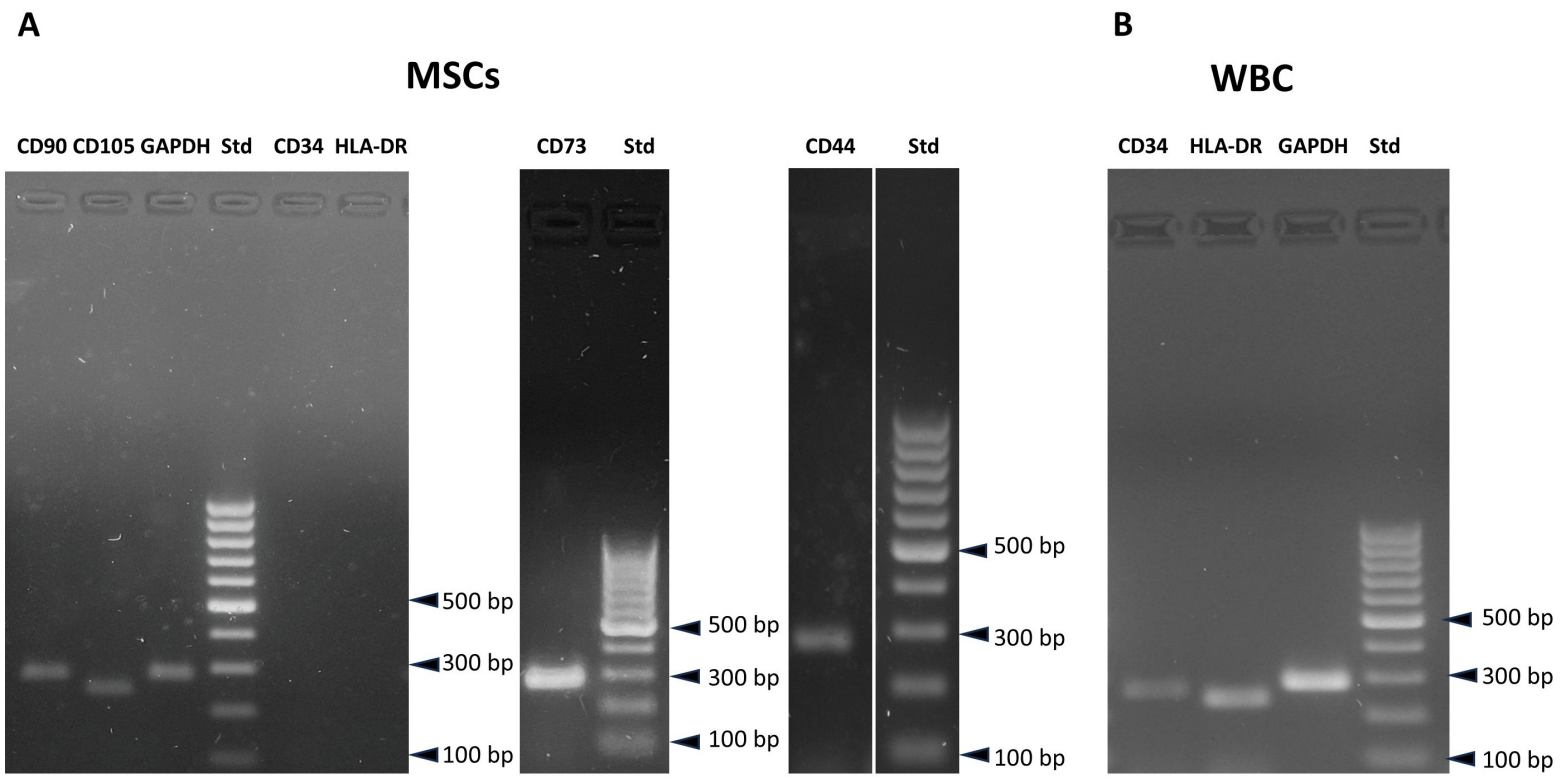
Gene expression analysis of *T. scripta* MSCs. MSCs are positive for CD90, CD73, CD105, and CD44 expression, while CD34 and HLA-DRA expression is not observed **(A)**. CD34 and HLA-DRA expression is positive in white blood cells **(B)**.

The identification of MSC populations in mammals typically relies on the analysis of gene expression for a panel of markers defined by the International Society for Cellular Therapy (ISCT) (30, 34). Given the unavailability of species-specific antibodies for *Trachemys scripta*, gene expression was analyzed using reverse transcription polymerase chain reaction (RT–PCR). Specific primers for the relevant markers were designed in accordance with the methodology outlined in the Materials and Methods. Polymerase chain reaction (PCR) was conducted on four distinct samples between passages P4 and P7, with three leukocyte samples serving as positive controls for markers anticipated to be negative in mesenchymal stem cells (MSCs). The resulting amplicons were subjected to sequencing to verify their alignment with known gene sequences in databases. For marker genes whose expression was not expected in MSCs (e.g., CD45, CD34, and HLA-DRA), expression was evaluated in white blood cell populations to validate the primers. The cells derived from adipose tissue and expanded in culture presented positive expression of CD90, CD105, CD44, and CD73, whereas CD34 and HLA-DRA expression was absent (Figure 7A). The presence of CD34 and HLA-DRA in white blood cells served to validate the accuracy of the primers (Figure 7B). The correspondence between the obtained sequences and the original sequences in the NCBI database was 100% for CD44, CD34, and CD73, 99% for CD105, CD90, and GAPDH, confirming that the obtained amplicons matched the expected sequences. The correspondence level of HLA-DR was 95% suggesting the possibility of natural polymorphisms or genetic diversity within the species. Amplicon sequences are reported in Supplementary material. CD45 expression was undetectable in both MSCs and leukocytes. CD31 was weakly expressed in MSCs, but sequencing did not confirm a match with the database sequences (data not shown). In this case, a possible discrepancy between the database sequence used for primer picking and the primers used is a potential explanation.

## 4 Discussion

In this study, MSC-like cells were isolated and expanded from the adipose tissue of red-eared sliders (*Trachemys scripta*) obtained during routine ovariosalpingectomy. The aim of this study was to evaluate the potential use of this species as a model for developing laboratory protocols to collect, isolate, expand, and characterize MSCs for future applications in other reptile species. We propose a method for adipose tissue collection that, while necessarily requiring a surgical procedure, can be considered minimally invasive for the animal. In these turtles, adipose deposits accumulate subcutaneously in the prefemoral region, which serves as the preferred access site for laparoscopic celiotomy procedures. This anatomical feature allows for efficient tissue sampling with minimal trauma (35). There is currently no detailed description in the literature of the structure of subcutaneous adipose deposits in the genus *Trachemys*. To confirm the nature of the collected adipose tissue, sample fragments were processed for histological analysis. Haematoxylin and eosin (H&E) staining revealed numerous clusters of mature adipocytes associated with fibrous tissue, blood vessels, lymphatics, and chromatophores (see Figure 2). The variable presence of chromatophores in the samples might be responsible for the brownish color typical of the adipose deposits collected. To evaluate the basic biological characteristics of the isolated cells, their cellular morphology, growth rate, ability to generate CFUs, trilineage differentiation capacity, and expression of a panel of gene markers suggested by the ISCT were analyzed (30). The protocol for *in vitro* isolation and expansion used in this study has been validated in mammals (36, 37) but not in reptiles. For example, reptiles are ectothermic animals (38); therefore, incubation parameters typically used for mammalian cells (37°C) are not applicable. To the best of our knowledge, to date, no studies have been published on the isolation, *in vitro* expansion, and identification of MSCs in this class of animals. However, studies in the literature have focused on toxicology involving fibroblast cultures from loggerhead turtles (*Caretta caretta*) (39, 40). Webb et al. (39) successfully isolated and characterized fibroblasts from the skin of loggerhead turtles. In their study, they analyzed various incubation temperatures for cultured cells (25, 30, 35°C) and demonstrated that 30°C was optimal for cell proliferation. Other studies suggest incubation temperatures between 26 and 30°C for marine turtle cell cultures (41). These data support the hypothesis that the ideal incubation temperature for cells, which allows for proliferation while minimizing stress and ensuring maximum survival during various passages, varies among not only different classes of animals but also different species. In the absence of validated protocols for obtaining MSCs from *Trachemys scripta* adipose tissue, we chose 28°C as the incubation temperature, which is the average value of the optimal thermal range for this species (41). It is also not surprising that this incubation temperature (28°C) has been used to expand cell cultures from other classes of ectothermic animals (amphibians and fish) (24, 42). Recently, a study by Otsuka-Yamaguchi et al. (24) demonstrated the feasibility of obtaining and characterizing MSC-like cells from the bone marrow of *Xenopus laevis*, an amphibian used as an experimental model in various fields of medicine owing to its ease of breeding, prolific nature, and excellent tissue regenerative capabilities (43). In the study by Otsuka-Yamaguchi et al., bone marrow was directly extracted from the bones of euthanized animals by mechanical fragmentation. In chelinators, bone marrow is contained within the spongy bone not only of the long bones and vertebrae but also of the carapace, bridge, and plastron (14, 44). Extracting bone marrow from these species therefore requires drilling into these structures to reach the bone cavities and access various cellular components. This procedure is invasive and painful, and it would also be challenging to obtain a sufficient sample size to isolate a significant number of MSCs for clinical application. Therefore, for ethical reasons, this method was excluded from this study. Instead, we evaluated the possibility of harvesting adequate quantities of adipose tissue during neutering surgeries. This approach was found to be feasible and not overly traumatic for the animals. We used two methods for cell isolation: collagenase digestion and mechanical fragmentation of adipose tissue followed by 3D culture. Both methods have been validated and described in mammals (28, 45, 46). Given the average size of a *Trachemys scripta*, it was not possible to obtain large amounts of adipose tissue from a single subject ( ≤ 1 g per subject). For the culture medium, DMEM was chosen because it is widely used for culturing MSCs in mammals, zebrafish (47), and loggerhead turtles (*Caretta caretta*) (39). Webb and colleagues evaluated different culture media (DMEM, MEM, and RPMI 1640) during the isolation and *in vitro* expansion of *Caretta caretta* fibroblasts and reported no statistically significant differences in cell growth among the various media (39). For this study, we also used DMEM supplemented with 20% fetal bovine serum (FBS), following the protocol used by Otsuka-Yamaguchi et al. (24) in *Xenopus laevis*. In terms of cell growth in culture, we observed a significant difference in the growth rate of the cells compared with those of mammals, fish, and amphibians. The average doubling time (DT) of the Ts-AD-MSCs at P3 was 127.2 h (5.3 days), which was greater than the DTs for bone marrow-derived MSCs from *Xenopus laevis* (25.6 h; 1.06 days) (24), MSCs isolated from the heart (50.67 h; 2.11 days) and liver (46.6 h; 1.94 days) of zebrafish (*Danio rerio*) at P4 (47), and adipose tissue MSCs from dogs (~36 h; 1.5 days at P3) (48–51). This difference could be related to the slower chelonian cellular metabolism (52) compared with those of other animal species, or it may be due to suboptimal culture conditions. However, no statistically significant differences were observed in the replicative potential of the cells from P1–P7, with both CDn and DT remaining constant across passages. In the *Xenopus laevis* study, a decrease in cellular replicative activity was noted from P5 onwards (24), whereas in zebrafish and dogs, this decrease was observed from P4 (47, 53). Several variables can influence the replicative capacity of stem cells in culture, such as the donor's age and health status (54, 55). The animals used in this study were provided by ENPA Parma, an animal protection organization, and were rescued from various situations (abandonment, found in parks and public places) and sterilized according to the Italian National Plan for the Management of the American Marsh Tortoise (*Trachemys scripta*). It was therefore not possible to determine the actual ages of the subjects, nor was it possible to estimate their ages accurately through morphometric investigations (56, 57). All the subjects included in the study were considered clinically healthy following a thorough clinical examination and routine hematological tests (58). However, reptiles with subclinical conditions may sometimes be asymptomatic or not show abnormalities in laboratory tests. Additionally, numerous variables (such as sex, breed, season, hormonal status, and captivity vs. wild state) can influence the normal reference values for each species (58). Therefore, it was not possible to definitively exclude underlying pathological conditions potentially affecting cell replication or other biological features. Cells were successfully differentiated into adipocytes, chondrocytes, and osteoblasts using differentiation medium validated for use with mammalian MSCs. This result is particularly interesting, as it demonstrates that Ts-AD-MSCs are responsive to the same differentiation factors. Similarly, in *Xenopus laevis* and *Danio rerio*, the differentiation of cells derived from the bone marrow (24), myocardium, and liver (47) has been shown to be positively influenced by chondrogenic, osteogenic, and adipogenic differentiation using human differentiation kits. With respect to the phenotypic characterization of the cells, it was not possible to use the validated method for mammalian MSCs, namely, flow cytometry (59), owing to the lack of validated antibodies for identifying *Trachemys scripta* CD markers. Consequently, the gene expression levels of various CD markers were assessed by RT–PCR, similar to the approach described by Otsuka-Yamaguchi et al. (24) for isolating MSCs from *Xenopus laevis* bone marrow. The *Trachemys scripta* genome was recently sequenced (32). To the best of our knowledge, no studies have measured the expression levels of the genes of interest for MSC characterization in the cells or tissues of the American pond turtle. Therefore, the primers used in this study were designed *de novo* based on gene sequences available on the NCBI website. The primer pairs used successfully amplified the genes of interest, with the exception of CD45 and CD31. Both the CD45- and CD31-encoding genes were investigated not only in MSCs but also in leukocytes isolated from blood, and the results were also negative. It is currently unclear whether the lack of expression of CD45 and CD31 is due to their actual absence in *T. scripta* leukocytes or analytical error. It is known from the literature that these genes are expressed in mammalian leukocytes, but there are no published studies regarding their expression in reptile leukocytes. Some recent studies have described CD45 expression in bird leukocytes (60, 61). As birds are phylogenetically close to reptiles (62, 63), this expression may be conserved. However, further studies are needed to verify its expression in this animal class. The cellular morphology, differentiation capacity into various mesenchymal cell types, and expression patterns of markers corresponding to mammalian features suggest that cells isolated from the adipose tissue of the flank of *Trachemys scripta* are likely to correspond to the mesenchymal stromal cell phenotype. Although some uncertainties remain concerning the results for CD45 and CD31, gene expression was substantially consistent with the ISCT expectations: the cells were positive for CD90, CD73, CD105, and CD44, whereas CD34 and HLA DRA expression was not observed.

We believe that our data suggest a substantial correspondence between the obtained cell populations and mammalian MSCs and support the hypothesis that applying regenerative medicine protocols in chelonian species will be possible in the future. Sea turtles belonging to various genera (e.g., *Caretta caretta, Chelonia mydas*, and *Dermochelys coriacea*) sustain significant traumatic injuries due to collisions with boat hulls or propellers. These injuries are frequently extensive and necessitate prolonged hospitalization, and some injured turtles ultimately cannot be released back into their natural environment (64). A similar fate affects thousands of terrestrial turtles worldwide each year, many of which are listed as “critically endangered” on the IUCN Red List (https://www.iucnredlist.org). Such injuries have been reported in wild turtles (65, 66) and in turtles kept as pets (67), including those injured by lawnmowers or other garden tools (67). The utilization of MSCs in such patients may prove advantageous, as evidenced by findings in other domestic animal species and humans.

The scientific literature on the use of regenerative medicine approaches for traumatic injuries in reptiles remains limited. Thrombocyte-leukocyte-rich plasma (TLRP) has been suggested as a treatment for traumatic injuries in various reptilian species, including chelonians, snakes, and lizards (7–9), with promising results. However, no data is available regarding MSC therapies. In contrast, an extensive body of research supports the application of MSCs in both soft and hard tissue injuries in mammals. In veterinary clinics, the use of biologics is gaining increasing interest for the treatment of both acute and chronic cutaneous lesions. MSCs have been shown to secrete bioactive factors that promote the proliferation of resident cells, facilitate extracellular matrix deposition and remodeling, enhance angiogenesis, and modulate the inflammatory response at the injury site, thereby accelerating wound healing (68, 69). Alongside soft tissue injuries, shell and bone fractures pose a significant threat to the survival of turtles and tortoises. In most chelonians, the carapace and plastron are structurally composed of both bone and dermal elements of the skin (70). Mesenchymal stem cells (MSCs) have been extensively studied for their differentiation potential toward the osteogenic lineage. However, their role in bone regeneration is likely mediated not only by direct differentiation but also through the secretion of immunoregulatory cytokines, the regulation of angiogenesis, and the modulation of the local microenvironment via trophic factor release (71, 72). Despite the absence of established clinical protocols for the use of MSCs in treating bone lesions in veterinary medicine, preliminary applications of cell-based therapies suggest promising indications of their safety and efficacy (73, 74). It can be hypothesized that, in the future, MSC-based therapies may also represent a viable treatment strategy for other vertebrates, including reptiles, complementing existing therapeutic approaches.

We are aware that the development of biological assays will be necessary to confirm that the population of MSC-like cells we have expanded maintains the key biological and therapeutic features of mammalian MSCs. Using biological assays to determine the potency of MSCs is crucial for evaluating their potential clinical application. Different assays have been proposed to assess the key functionalities of MSCs used in veterinary medicine, mainly aimed at demonstrating immunomodulation and tissue regeneration ability (6, 34). Future studies will be necessary to optimize standardized protocols, including cell sources, culture methods, passage numbers, and clinical handling protocols.

## 5 Conclusion

The results of the present study suggest that it is feasible to isolate and expand *in vitro* populations of MSC-like cells harvested from turtles starting from small samples of adipose tissue. Despite the absence of scientific literature demonstrating a functional correspondence between these cells and those isolated from mammals, which have been extensively studied for their immunomodulatory and proregenerative properties, the present work paves the way for further studies aimed at the application of regenerative medicine protocols in Chelonians.

## Data Availability

The datasets presented in this study can be found in online repositories. The names of the repository/repositories and accession number(s) can be found in the article/Supplementary material.
