# Supplementary material for: Mesenchymal stromal cell isolation from pond slider (Trachemys scripta) adipose tissue obtained during routine neutering: a model for turtle species
Source: Front Vet Sci. 2025 Mar 19;12:1546091. doi: 10.3389/fvets.2025.1546091 (PMC11963382; doi:10.3389/fvets.2025.1546091)
Supplement: Supplementary file 1 [file Data_Sheet_1.pdf]

Sequence analysis of amplicons obtained by RT-PCR.

Sequence omology evaluation was performed using BLASTn to validate the expression of a set of genes used for the phenotypic characterization of MSCs from *Trachemys Scripta*.

Gene expression analysis was performed for:

Gene	NCBI Reference Sequence	Expressed in
CD44	XM_034769691.1	MSCs
CD34	XM_034770555.1	white blood cells
CD73	XM_034766097.1	MSCs
CD90	XM_034754732.1	MSCs
CD105	XM_034793487.1	MSCs
HLA-DR	XM_034789225.1	white blood cells
GAPDH	XM_034787619.1	MSCs, white blood cells

Sequence analysis online tool: https://blast.ncbi.nlm.nih.gov/Blast.cgi

CD44

Query	XM_034769691.1
Subject	RT-PCR amplicon
% identity	100%
E-value	7e-118

```
Query: PREDICTED: Trachemys scripta elegans CD44 molecule (Indian blood group) (CD44), mRNA Query ID: XM_034769691.1
Subject: RT-PCR amplicon gene: CD44

Score:409 bits(221), Expect:7e-118,
Identities:221/221(100%),  Gaps:0/221(0%), Strand: Plus/Minus

Query  2731  GCTTCTACAGCCACCAGTTCTACACTTAACAAACACGATGGCAGAAGAGCAAATCCATCT  2790
             ||||||||||||||||||||||||||||||||||||||||||||||||||||||||||||
Sbjct  222   GCTTCTACAGCCACCAGTTCTACACTTAACAAACACGATGGCAGAAGAGCAAATCCATCT  163

Query  2791  GGACAGGTCACAACACCAAAAACAGCTTCACAGCCTAGATCAGCCAAAATACCAGAATGG  2850
             ||||||||||||||||||||||||||||||||||||||||||||||||||||||||||||
Sbjct  162   GGACAGGTCACAACACCAAAAACAGCTTCACAGCCTAGATCAGCCAAAATACCAGAATGG  103

Query  2851  CTGATCATAGTGGCTTCTCTTCTGGCACTGACATTGATTCTGGCAGTCTGCATTGCTGTC  2910
             ||||||||||||||||||||||||||||||||||||||||||||||||||||||||||||
Sbjct  102   CTGATCATAGTGGCTTCTCTTCTGGCACTGACATTGATTCTGGCAGTCTGCATTGCTGTC  43

Query  2911  AACAGCCGGAGCAGATGTGGGCAGAAGAAAAAGCTAGTGAT  2951
             |||||||||||||||||||||||||||||||||||||||||
Sbjct  42    AACAGCCGGAGCAGATGTGGGCAGAAGAAAAAGCTAGTGAT  2
```

CD34

Query	XM_034770555.1
Subject	RT-PCR amplicon
% identity	100
E-value	1e-116

```
Query: PREDICTED: Trachemys scripta elegans CD34 molecule (CD34), transcript variant X1, mRNA Query ID: XM_034770555.1


>
Sequence ID: Query_1260807 Length: 218
Range 1: 1 to 218

Score:403 bits(218), Expect:1e-116,
Identities:218/218(100%),  Gaps:0/218(0%), Strand: Plus/Minus

Query  1035  TGCAGAGTATTGAACGCCACCAGAACATTCCCCGGAACACGCTGATTGCCTTGGTCACAT  1094
             ||||||||||||||||||||||||||||||||||||||||||||||||||||||||||||
Sbjct  218   TGCAGAGTATTGAACGCCACCAGAACATTCCCCGGAACACGCTGATTGCCTTGGTCACAT  159

Query  1095  CTGGACTCTTGCTGGCGTTTCTGAGTTTGGCTGGATACTTCCTTATGAAACGACGGAGTT  1154
             ||||||||||||||||||||||||||||||||||||||||||||||||||||||||||||
Sbjct  158   CTGGACTCTTGCTGGCGTTTCTGAGTTTGGCTGGATACTTCCTTATGAAACGACGGAGTT  99

Query  1155  GGAGCCCCAGGGGAGAGAGGCTGGATGAAGACCTCTATTACACTGAAAACGGTAGCCAGG  1214
             ||||||||||||||||||||||||||||||||||||||||||||||||||||||||||||
Sbjct  98    GGAGCCCCAGGGGAGAGAGGCTGGATGAAGACCTCTATTACACTGAAAACGGTAGCCAGG  39

Query  1215  GAAATACAGGGATCACCGTGGCCTCCCAAGAGCACTCC  1252
             ||||||||||||||||||||||||||||||||||||||
Sbjct  38    GAAATACAGGGATCACCGTGGCCTCCCAAGAGCACTCC  1
```

CD73

Query	XM_034766097.1
Subject	RT-PCR amplicon
% identity	100%
E-value	2e-134

```
Query: PREDICTED: Trachemys scripta elegans 5'-nucleotidase ecto (NT5E), transcript variant X2, mRNA Query ID: XM_034766097.1


>
Sequence ID: Query_6143783 Length: 315
Range 1: 21 to 276

Score:462 bits(512), Expect:2e-134,
Identities:256/256(100%),  Gaps:0/256(0%), Strand: Plus/Minus

Query  1418  GGAAATTTGATTTGTGATGCCATGATTTACAATAATCTCAAACATCGAGATGAAAATACA  1477
             ||||||||||||||||||||||||||||||||||||||||||||||||||||||||||||
Sbjct  276   GGAAATTTGATTTGTGATGCCATGATTTACAATAATCTCAAACATCGAGATGAAAATACA  217

Query  1478  TGGAATCATGTTTCAATGTGCATCATAAATGGCGGAGGGATACGGTCACCCATTGATGAA  1537
             ||||||||||||||||||||||||||||||||||||||||||||||||||||||||||||
Sbjct  216   TGGAATCATGTTTCAATGTGCATCATAAATGGCGGAGGGATACGGTCACCCATTGATGAA  157

Query  1538  CGCAACAATAATGGTACTATTACGATGGAGGACCTGCTAGCTGTGCTGCCATTTGGAAGT  1597
             ||||||||||||||||||||||||||||||||||||||||||||||||||||||||||||
Sbjct  156   CGCAACAATAATGGTACTATTACGATGGAGGACCTGCTAGCTGTGCTGCCATTTGGAAGT  97

Query  1598  ACTTTTGATCTGATTGAGTTAAAAGGCTCCACTCTCAAAGAAGCATTTGAGCATGGTGTG  1657
             ||||||||||||||||||||||||||||||||||||||||||||||||||||||||||||
Sbjct  96    ACTTTTGATCTGATTGAGTTAAAAGGCTCCACTCTCAAAGAAGCATTTGAGCATGGTGTG  37

Query  1658  CGCAGACATGGACAAG  1673
             ||||||||||||||||
Sbjct  36    CGCAGACATGGACAAG  21
```

CD90

Query	XM_034754732.1
Subject	RT-PCR amplicon
% identity	99
E-value	1e-124

```
Query: PREDICTED: Trachemys scripta elegans Thy-1 cell surface antigen (THY1), mRNA Query ID: XM_034754732.1

Subject: CD90 amplicon
Sequence ID: Query_3807515 Length: 242
Range 1: 1 to 239

Score:431 bits(233), Expect:1e-124,
Identities:237/239(99%),  Gaps:0/239(0%), Strand: Plus/Minus

Query  364  GAACAGCCTCAAGACCCGATCCAACATCACCCTGTCCAAGGACCTGGCGTGCCTGCAGCT  423
            ||||||||||||||||||||||||||||||||||||||||||||||||||||||||||||
Sbjct  239  GAACAGCCTCAAGACCCGATCCAACATCACCCTGTCCAAGGACCTGGCGTGCCTGCAGCT  180

Query  424  GTTCGACTTCACCACCGCAGACGAGGGCATCTACATCTGCGAGCTGAAGATCACCGGTGA  483
            ||||||||||||||||||||||||||||||||||||||||||||||||||||||||||||
Sbjct  179  GTTCGACTTCACCACCGCAGACGAGGGCATCTACATCTGCGAGCTGAAGATCACCGGTGA  120

Query  484  CTACACCGGCAACCAGATAAGGAACATCACCGTCATCAAAGACAAGCTGGAGAAATGTGC  543
            ||||||||||||||||||||||||||||||||||||||||||||||||||||||||||||
Sbjct  119  CTACACCGGCAACCAGATAAGGAACATCACCGTCATCAAAGACAAGCTGGAGAAATGTGC  60

Query  544  TGGCATCAGCCTCTTGATTCAGAACACTTCCTGGTTGCTGCTCCTGCTCCTGTCTCTGC  602
            |||||||||||||||||||||||||||||||||||||||||||||||||||||  ||||
Sbjct  59   TGGCATCAGCCTCTTGATTCAGAACACTTCCTGGTTGCTGCTCCTGCTCCTGTGCCTGC  1
```

CD105

Query	XM_034793487.1
Subject	RT-PCR amplicon
% identity	99
E-value	2e-103

```
Query: PREDICTED: Trachemys scripta elegans endoglin (ENG), mRNA Query ID: XM_034793487.1
Subject: CD105 amplicon

Sequence ID: Query_710297 Length: 212
Range 1: 6 to 209

Score:361 bits(195), Expect:2e-103,
Identities:201/204(99%),  Gaps:0/204(0%), Strand: Plus/Plus

Query  1467  GAACTTACAGGATTTAAATGACTACATCACAGATATCACATTGCAAGACCCCCGTTGCAG  1526
             |||||||||||||||||||||||||||||| ||| |||||||||||||||||||||||||
Sbjct  6     GAACTTACAGGATTTAAATGACTACATCACGGATGTCACATTGCAAGACCCCCGTTGCAG  65

Query  1527  AGCTGAACAAAACCAAACTCACTTTGTGCTGAGAAGGTATTTGGGGGACTGCCACACCAA  1586
             ||||||||||||||||||||||||||||||||||||||||||||||||||||||||||||
Sbjct  66    AGCTGAACAAAACCAAACTCACTTTGTGCTGAGAAGGTATTTGGGGGACTGCCACACCAA  125

Query  1587  ACTGGAGAGTGACATTCTAGCCATGAATAAGCTGATCCTGACACTGGCTTCATCGCTGGA  1646
             ||||||||||||||||||||||| ||||||||||||||||||||||||||||||||||||
Sbjct  126   ACTGGAGAGTGACATTCTAGCCAGGAATAAGCTGATCCTGACACTGGCTTCATCGCTGGA  185

Query  1647  GAAAGTTGAGGTGCCGTTCGAGTG  1670
             ||||||||||||||||||||||||
Sbjct  186   GAAAGTTGAGGTGCCGTTCGAGTG  209
```

HLA-DR

Query	XM_034789225.1
Subject	RT-PCR amplicon
% identity	95
E-value	4e-90

```
Query: PREDICTED: Trachemys scripta elegans HLA class II histocompatibility antigen, DR alpha chain-like (LOC117886999), transcript variant X1, mRNA Query ID: XM_034789225.1
Length: 1525

Subject: HLA-DR amplicon
Sequence ID: Query_7452125 Length: 206
Range 1: 5 to 201

Score:315 bits(170), Expect:4e-90,
Identities:189/198(95%),  Gaps:1/198(0%), Strand: Plus/Minus

Query  620   AGGGCGACTTCTACGACTGCCGGGTGGAGCACTGGGGGCTGCCCGAGCCCTTCACGAAGC  679
             ||||||||||||||||||||| |||||||||| |||||||||||||||||||||||||||
Sbjct  201   AGGGCGACTTCTACGACTGCCAGGTGGAGCACGGGGGGCTGCCCGAGCCCTTCACGAAGC  142

Query  680   ACTGGGAAGTCCAGGTGCCCACCCCTGTCCCCAAGACCTCAGAGACCCTGGTGTGCGCCC  739
             ||||||||||||||||||||||||| |||||| ||||| |||||||||||||||||||||
Sbjct  141   ACTGGGAAGTCCAGGTGCCCACCCCCGTCCCCGAGACCACAGAGACCCTGGTGTGCGCCC  82

Query  740   TGGGCCTGGCCGTGGGCATCATCGGCATCATCGTGGGCACCATCCTCATCATCAAGGGCA  799
             ||||||||||||||||||||||||||||||||| |||||||||||||||||||||||| |
Sbjct  81    TGGGCCTGGCCGTGGGCATCATCGGCATCATCGCGGGCACCATCCTCATCATCAAGGGGA  22

Query  800   TGAAGATGAACGCCGCCC  817
             |||||| ||||||| |||
Sbjct  21    TGAAGA-GAACGCCCCCC  5
```

GAPDH

Query	XM_034787619.1
Subject	RT-PCR amplicon
% identity	99
E-value	1e-131

```
Query: PREDICTED: Trachemys scripta elegans glyceraldehyde-3-phosphate dehydrogenase (GAPDH), mRNA Query ID: XM_034787619.1
Subject: GAPDH amplicon

Sequence ID: Query_3536427 Length: 252
Range 1: 2 to 249

Score:453 bits(245), Expect:1e-131,
Identities:247/248(99%),  Gaps:0/248(0%), Strand: Plus/Plus

Query  856   CTCACAATGTCTCTGTCGTGGACCTGACTTGTCGTCTGGAAAAACCAGCCAAGTATGATG  915
             ||||||||||||||||||||||||||||||||||||||||||||||||||||||||||||
Sbjct  2     CTCACAATGTCTCTGTCGTGGACCTGACTTGTCGTCTGGAAAAACCAGCCAAGTATGATG  61

Query  916   ACATTAAGAAGGTGATGAAAAGTGCCTCTGAGGGGCCTATGAAGGGCATCCTGGGATACA  975
             ||||||||||||||||||||||||||||||||||||||||||||||||||||||||||||
Sbjct  62    ACATTAAGAAGGTGATGAAAAGTGCCTCTGAGGGGCCTATGAAGGGCATCCTGGGATACA  121

Query  976   CAGAGGACCAGGTTGTCTCCTGTGACTTCAACGGCGACAGCCACTCATCCATCTTTGATG  1035
             ||||||||||||||||||||||||||||||||||||||||||||||||||||||||||||
Sbjct  122   CAGAGGACCAGGTTGTCTCCTGTGACTTCAACGGCGACAGCCACTCATCCATCTTTGATG  181

Query  1036  CAGCTGCTGGCATTGCTCTCAATGATCACTTTGTCAAACTGGTCTCCTGGTATGACAATG  1095
             ||||||||||||||||||||||||||||||||||||||||||||||||||||||| ||||
Sbjct  182   CAGCTGCTGGCATTGCTCTCAATGATCACTTTGTCAAACTGGTCTCCTGGTATGAAAATG  241

Query  1096  AGTTTGGA  1103
             ||||||||
Sbjct  242   AGTTTGGA  249
```
